# The Microbiota–Gut–Brain Axis in Depression: Mechanisms, Microbiota‐Targeted Interventions, and Translational Challenges

**DOI:** 10.1155/ijm/6750078

**Published:** 2025-12-23

**Authors:** Liang Zeng, Siyu Zhang, Ruoxi Liu, Lili Wang, Yurong Tan

**Affiliations:** ^1^ Department of Medical Microbiology, Xiangya School of Basic Medicine, Central South University, Changsha, Hunan, China, csu.edu.cn

**Keywords:** depression, intestinal dysbiosis, microbiota–gut–brain axis, probiotics

## Abstract

Depression, a global mental health pandemic, persists with unmet therapeutic needs due to the limitations of conventional antidepressants. Emerging evidence suggests that the microbiota–gut–brain axis (MGB axis) is a crucial regulator of depressive pathophysiology, facilitating bidirectional communication between the gut microbiota and the central nervous system (CNS) through neural, immune, endocrine, and metabolic pathways. This review explores the complex mechanisms underlying MGB dysfunction in depression, including vagus nerve‐mediated signaling, cytokine‐driven neuroinflammation, and hypothalamic–pituitary–adrenal (HPA) axis dysregulation. Innovations in microbiota‐targeted interventions, ranging from probiotic engineering and precision dietary modulation to bacteriophage therapy and AI‐driven personalized medicine, have been critically assessed for their potential to restore MGB homeostasis. By linking mechanistic insights with clinical translation, this work outlines a roadmap for transforming the gut microbiota into a therapeutic frontier for depression.

## 1. Introduction

### 1.1. The MGB Axis

**Figure 1:**
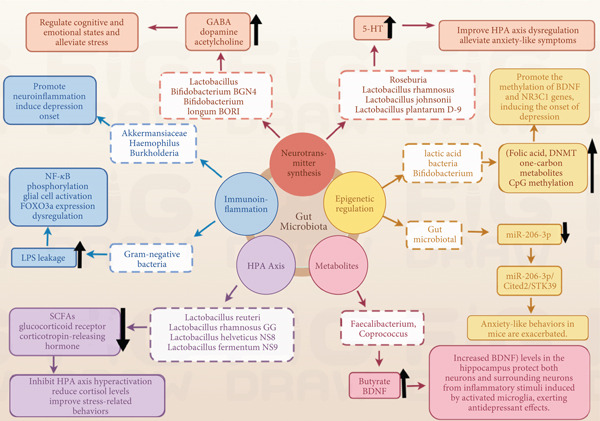
Functional pathways of the MGB axis.

The MGB axis allows the gut microbiota to modulate CNS function via interconnected signaling networks. In the neural pathway, the vagus nerve transmits intestinal signals to brain regions; vagotomy reduces probiotic efficacy for social deficits and anxiety [1–5], highlighting its role. The enteric nervous system (ENS) acts as the “second brain,” managing intestinal homeostasis; dysfunction causes permeability and neuroinflammation, impacting neuroplasticity [1]. In the immune pathway, two key barriers maintain homeostasis: the blood–brain barrier (BBB) and intestinal epithelial barrier (IEB). Disruption of the IEB allows harmful substances (e.g., lipopolysaccharide (LPS)) to enter the bloodstream. This increases BBB permeability and triggers proinflammatory cytokines, which contribute to depressive phenotypes [6]. Gut microbiota orchestrate immunity, with GF animals showing impairment [7, 8]. Stress or dysbiosis compromises barriers, activating TLR4/NF‐*κ*B pathways and promoting inflammation [9]. For the endocrine pathway, probiotics enhance behavior and regulate the HPA axis [10, 11]. Chronic stress initiates cycles of intestinal barrier leakage (i.e., increased permeability of the IEB, allowing LPS and bacterial metabolites to translocate into the systemic circulation) and neuroinflammation [12], while dysbiosis induces anxiety and HPA hyperactivity [13–15]; microbial dysregulation activates the HPA via IL‐1*β* and LPS [16]. Regarding metabolites, short‐chain fatty acids (SCFAs) strengthen BBB integrity and engage microglia [17]. Microbiota regulate serotonin production and synthesize GABA, reducing anxiety [18–21] (Table 1 and Figure 1).

**Table 1:** Mechanisms of cross‐regulation of neural, immune, and endocrine pathways.

**Pathway**	**Key mechanism**	**Interaction with other pathways**	**Related metabolites/factors**	**Intervention strategies**
Neural pathway	The vagus nerve and ENS	Immune‐inflammatory signals activate central neuroinflammation via the vagus nerve; cortisol from the HPA axis inhibits hippocampal neuroplasticity.	5‐HT, GABA, BDNF, vagal neurotransmitters	Probiotics such as *L. rhamnosus* activate the vagus nerve. Psychobiotics regulate neurotransmitter synthesis.
Immune pathway	Cytokines and immune cells	Inflammatory cytokines induce neuroinflammation through the BBB; pro inflammatory cytokines activate the HPA axis.	LPS, IL‐6, TNF‐*α*, TLR4/NF‐*κ*B pathway	Dietary interventions (high‐fiber/anti‐inflammatory diets) repair the intestinal barrier. Antibiotics such as minocycline inhibit pro‐inflammatory bacteria.
Endocrine pathway	Hyperactivity of the HPA axis → sustained elevation of cortisol →exacerbation of intestinal leakage; early microbial colonization programs the sensitivity of the HPA axis.	Cortisol inhibits the function of ENS and tight junction proteins of the intestinal barrier; immune‐inflammatory factors enhance the stress response of the HPA axis.	Cortisol, CRH, glucocorticoid receptor (GR), BDNF	Probiotics such as *L. helveticus* NS8 inhibit hyperactivity of the HPA axis. Early supplementation with Bifidobacterium regulates the HPA axis.
Metabolic pathway	Reduced synthesis of SCFAs and decreased intestinal barrier repair capacity; Tryptophan metabolism shifts toward the kynurenine pathway	SCFAs enhance neuroplasticity by inhibiting HDAC; kynurenine metabolites induce neurotoxicity and activate microglia.	SCFAs, tryptophan, kynurenine, GABA	A high‐fiber diet promotes the production of SCFAs. Probiotics such as *L. plantarum D-9* regulate tryptophan metabolism.

### 1.2. Microbiome Dysregulation in Depression: Markers and Mechanisms

The human gut microbiota primarily comprises *Firmicutes* and *Bacteroidetes* [22], with others like Proteobacteria. Extrinsic factors, such as diet, environmental pollutants, medication exposure, and lifestyle habits, can profoundly disturb the composition and functional balance of gut microbiota. These disturbances often lead to immediate consequences including intestinal dysbiosis, compromised IEB function, and altered microbial metabolite synthesis—all of which contribute to the pathological processes underlying depression. Restoring eubiosis, or a stable and balanced gut microbial ecosystem, has therefore emerged as a critical target for mitigating microbiota‐associated depressive phenotypes [23]. In major depressive disorder (MDD) patients, microbiota shows abnormalities in composition, diversity, and function, linked to neuroinflammation, metabolic disorders, and behavioral abnormalities. MDD patients exhibit reduced microbiota diversity, with decreased *Firmicutes* abundance and fewer butyrate‐producing genera like *Faecalibacterium*, weakening SCFA production and anti‐inflammatory functions [24]. Conversely, *Bacteroidetes* abundance increases, activating microglia and promoting systemic inflammation, correlating with disease severity [25]. Probiotics like *Lactobacillus* and *Bifidobacterium* are reduced, associated with lower BDNF and impaired serotonin synthesis, while proinflammatory bacteria such as *Enterobacteriaceae* are elevated [26, 27]. In contrast, proinflammatory bacteria such as Enterobacteriaceae are elevated [26]. Fecal calprotectin (a marker of intestinal inflammation) is decreased in emotion‐regulating brain regions like the superior frontal gyrus—contrasting with healthy individuals [27]. Metabolic dysfunction is prevalent, with reduced SCFAs damaging the gut barrier, increasing permeability and allowing LPS entry, causing inflammation and depression‐like behavior [27]. Dysbiosis promotes gram‐negative bacteria growth, releasing LPS and triggering inflammatory storms that harm hippocampal synapses [28].

## 2. Revolutionizing Treatment: Microbiota‐Targeted Innovations

**Figure 2:**
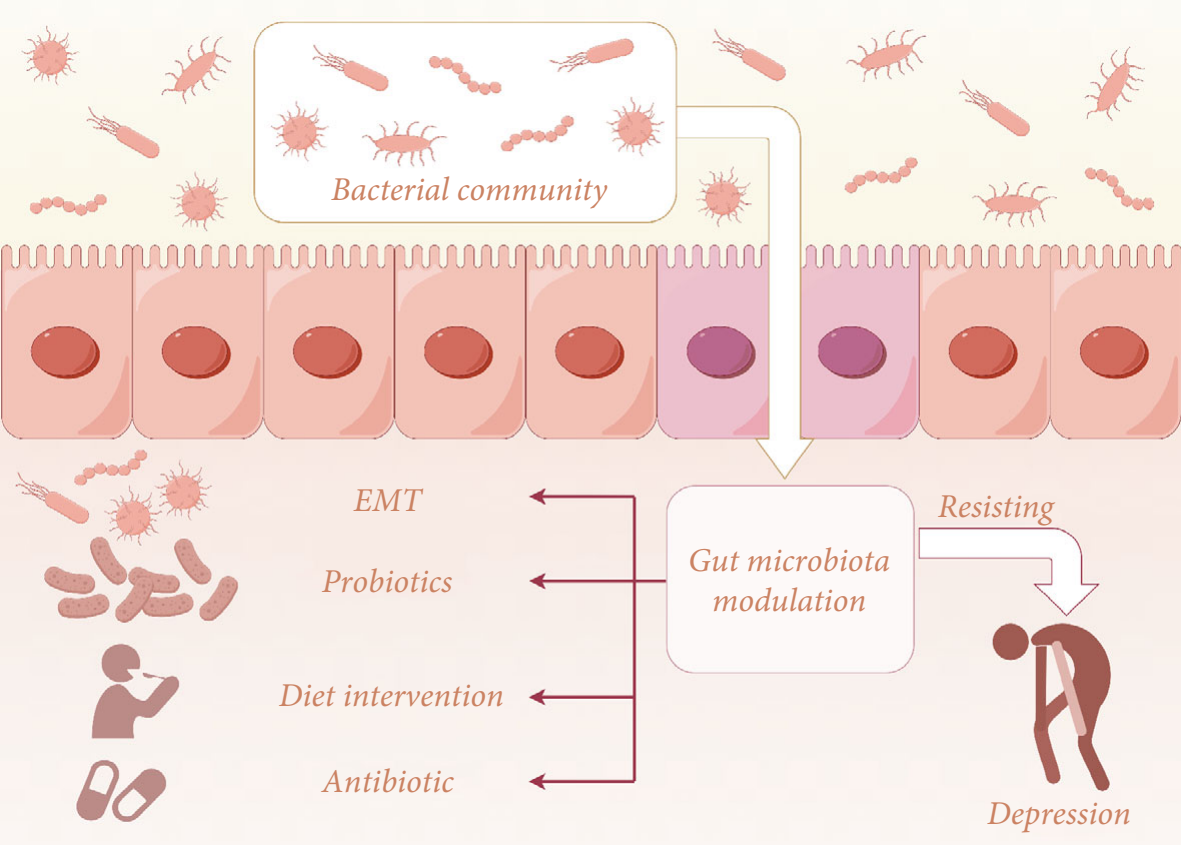
Microbiota‐targeted treatment.

Although traditional antidepressant therapies, such as selective serotonin reuptake inhibitors (SSRIs), are widely used, they have significant limitations: approximately 30%–40% of patients do not respond to these drugs, and common gastrointestinal side effects, such as nausea and constipation, as well as the risk of metabolic disorders, are prevalent. In contrast, intervention strategies targeting the gut microbiota offer an innovative approach to treating depression by modulating the gut–brain axis through multiple pathways, including neurological, immune, and metabolic ones. The core advantages of these strategies are as follows: (1) their multitarget role, such as the regulation of inflammation and neurotransmitter synthesis; (2) reduced drug dependence; and (3) noninvasive methods, such as diet or probiotics, to improve treatment compliance [29]. The following section will analyze specific experimental studies based on the intestinal microbiota to discuss appropriate antidepressant strategies and their safety and feasibility (Figure 2).

### 2.1. Probiotics: From Strains to Smart Microbes

Probiotics enhance verbal episodic memory in depressed patients by increasing intestinal *Lactobacillus* and regulating hippocampal activation [30, 31]. A multicenter, double‐blind RCT by Nikolova et al. (*n* = 240) showed that adjunctive probiotics (*L. rhamnosus* + *B. longum*) improved HAM‐D scores in 52% of MDD patients, with low dropout rates (12%)—supporting clinical feasibility [30]. However, a smaller trial (*n* = 60) found no significant effects, likely due to unstratified enrollment (mixing high/low inflammation subgroups) and short duration (4 vs. 8 weeks) [31]. *Akkermansia muciniphila*, a next‐generation probiotic, reduces depressive‐like behaviors in mice by thickening the intestinal mucus layer and modulating SCFAs, with anti‐inflammatory and neuroprotective effects. Its modified outer membrane protein Amuc_1100*Δ*80 interacts with intestinal epithelial TLR2 to increase intestinal 5‐HT, alleviating chronic unpredictable mild stress (CUMS)–induced anxiety and depression more potently than the original protein [32]. In a mouse alcohol–LPS model, *A. muciniphila* improved alcohol‐related depression by strengthening the intestinal barrier, reducing serum LPS, mitigating neuroinflammation (lower TNF‐*α*/IL‐1*β*), normalizing depression‐related gene expression, and increasing hippocampal 5‐HT [33], supporting its potential to alleviate depressive symptoms. Precision probiotic interventions target specific populations: A randomized trial showed *L. reuteri* PBS072 and *B. breve* BB077 significantly improved postpartum depression (EPDS scores) by enhancing stress recovery [34].

Engineered *Escherichia coli* Nissle 1917 secreting BDNF reversed depression‐like behaviors in animals [35]. These strains use “smart” circuits—for example, *E. coli* engineered with a tryptophan‐inducible promoter produces 5‐HTP only in the gut (where tryptophan is abundant), avoiding off‐target neurochemical effects [36]. AI‐driven personalized approaches use wearable devices to monitor SCFAs and guide probiotic dose adjustments [37]. Postbiotics (inanimate microbial components/metabolites) offer new options: Heat‐killed *Lactobacillus plantaris*‐derived postbiotics, via metabolites like SCFAs and bile acids, protect against *Salmonella*‐induced depression by modulating the gut–brain axis [38].

However, probiotic efficacy varies due to baseline microbiota differences (e.g., patients with low *Faecalibacterium* respond better [39]). Engineered strains carry bacteremia risks in immunocompromised hosts, requiring strict preclinical safety testing (e.g., assessing bacterial translocation in immunodeficient mice [36]).

### 2.2. Dietary Precision: Fiber, Polyphenols, and Personalization

High‐fiber diets relieve neuroinflammation by boosting SCFA production via microbial fermentation. SCFAs regulate microglia, inhibit hippocampal NF‐*κ*B, and reduce proinflammatory cytokines, improving chronic stress‐induced depression in animals [40]. Unlike medications linked to metabolic syndrome, they enhance insulin sensitivity and reduce obesity‐related depression risk via SCFAs like propionate [31]. The PREDITAP trial showed a Mediterranean diet (rich in polyphenols/Omega‐3s) reduced depression risk in adolescents and adults, with HAM‐D scores decreasing by 2.15 (4 weeks) and 2.42 (8 weeks, *p* < 0.05), linked to increased microbiota diversity [40]. Polyphenols (e.g., resveratrol) strengthen the intestinal barrier and inhibit systemic inflammation (20% lower C‐reactive protein (CRP)), differing from traditional calorie‐focused diets [40]. However, cross‐sectional studies report inconsistent associations, attributed to self‐reported diet recall bias [41]. Ketogenic diets (high‐fat, low‐carb) improve depression/anxiety in animal models but face clinical challenges: small, uncontrolled studies, short‐term focus, high dropout rates, and methodological heterogeneity [42]. Anti‐inflammatory diets (low processed foods, high fruits/vegetables/whole grains) reduce depression scores in patients with elevated IL‐6/TNF‐*α*. Curcumin and flavonoids (e.g., blueberry anthocyanins) inhibit microglial activation, making them potential options for inflamed subgroups [43].

However, personalized dietary plans consider gene‐nutrition interactions: 5‐HTTLPR s‐allele carriers are sensitive to high‐sugar diets, with low‐carb diets reducing depression risk [44]; FTO gene mutants benefit more from low‐GI diets [45]. Strict diets like ketogenic regimens have poor long‐term adherence, requiring adaptable modifications. Nonresponders need microbiome/metabolome stratification.

### 2.3. FMT and Beyond: From Stool Transplant to Synthetic Microbiomes

Fecal microbiota transplantation (FMT) regulates gut flora to treat depression. Transplanting healthy donor microbiota into depressed mice reversed behavioral abnormalities—reducing forced swimming immobility by 50% [46]. It also increased levels of the anti‐inflammatory bacterium *Faecalibacterium prausnitzii*. These effects are mediated by vagal signaling and HPA axis normalization [46]. Preliminary trials show FMT improves gastrointestinal symptoms and quality of life in treatment‐resistant depression (TRD), but efficacy varies: one case report noted HAM‐D reduction from 24 to 14 (8‐week follow‐up), but small sample size (*n* = 15) and lack of long‐term data limit generalizability [47]. FMT combined with SSRIs accelerates remission (2 weeks earlier than monotherapy) by restoring microbiota–tryptophan metabolism for serotonin synthesis [46].

However, FMT faces microbiota “reversion” (60% of patients return to baseline flora by 12 months), increasing metabolic syndrome risk (OR = 1.8) [48]. Donor selection, transplantation routes (oral vs. colonoscopy), and ethics limit clinical use [48]. FMT′s potential requires addressing donor screening standardization, long‐term safety, and individual heterogeneity. Future directions include synthetic biology‐modified microbiota and multiomics‐guided personalization.

### 2.4. Potential Use of Antibiotics

Anti‐inflammatory antibiotics like minocycline and doxycycline show promise as adjuncts for depression, with neuroprotective and flora‐regulating properties. Some SSRIs (e.g., fluoxetine) also exhibit antibiotic activity. Notably, antibiotics do not exert uniform effects on gut microbiota; their impact varies significantly based on factors such as antibiotic class, administration dose, treatment duration, and the initial composition of the gut microbial community. While certain antibiotics may reduce the abundance of pro‐inflammatory bacteria, others can disrupt beneficial microbial populations, potentially exacerbating gut dysbiosis if not used strategically [49]. A meta‐analysis confirmed minocycline′s benefit in unipolar depression (especially psychotic subtypes) but not bipolar disorder [50]. An RCT found depressed patients with low‐grade inflammation (CRP ≥ 3 mg/L) had a 2.5‐point greater HAM‐D reduction with 4‐week minocycline versus placebo (*p* = 0.03) [51]. Doxycycline exerts neuroprotective, anti‐inflammatory, and antidepressant effects, restoring LPS‐induced behavioral/neuroinflammatory responses in mice by inhibiting stress‐induced NO synthesis and iNOS [52]. Selective antibiotics like rifaximin (nonsystemic, gut‐targeted) reduce inflammation/oxidative stress and improve depression by inhibiting ammonia‐producing bacteria (e.g., *Klebsiella*). It modulates gut flora and tryptophan metabolism to alleviate CUMS‐induced depression in rats [53].

However, broad‐spectrum antibiotics (e.g., against Gram‐negative bacteria) reduce flora diversity and risk resistance [54]. Short‐term (≤ 4 weeks) use with probiotics (e.g., *Bifidobacterium*) restores 90% flora diversity and reduces drug‐resistant bacteria, limiting use to infection‐associated depression subtypes with concurrent probiotic support [55]. Long‐term broad‐spectrum use causes dysbiosis, resistance, opportunistic infections (e.g., *C. difficile*), and metabolic disorders. Their use requires strict limitation to inflammatory subtypes, with flora monitoring.

### 2.5. Phage Therapy

Phages regulate gut flora homeostasis with strain‐specific targeting of pathogens, sparing beneficial bacteria. Metagenomics show MDD patients have imbalanced phage communities: reduced *Clostridium bacteriophage* phi8074‐B1 and *Klebsiella bacteriophage* vB‐KpnP‐SU552A (50%–70%), and increased *Escherichia bacteriophage* ECBP5 (2.3×) [56], disrupting phage–host symbiosis to promote pathogenic overgrowth, intestinal permeability, and neuroinflammation.

In chronic restraint stress‐induced depressed mice, *Enterobacteriaceae, Gammaproteobacteria*, and *Campylobacteraceae* phages are enriched, targeting proinflammatory *Enterobacteriaceae* [57]. Tryptophan metabolites (tryptamine and 5‐methoxytryptamine) correlate with Microviridae and Podoviridae phages, suggesting phages influence serotonin synthesis via bacterial tryptophan metabolism enzymes [57].

Phages modulate the MGB axis via dual pathways: (1) lysing proinflammatory Enterobacteriaceae to reduce LPS‐induced neuroinflammation and (2) altering bacterial tryptophan metabolism—for example, increasing *Bifidobacterium* abundance (via *Clostridium* phages) to boost 5‐HT synthesis [57]. Engineered phages could carry “therapeutic genes” (e.g., SCFA synthase) to restore microbiota function—addressing the limitation of natural phages (which only lyse pathogens, not restore beneficial metabolites).

No human trials for depression exist, but phage therapy for gut infections (e.g., *C. difficile*) has shown safety in Phase I trials (*n* = 50)—supporting translatability [56]. An upcoming pilot trial (NCT05876321) will test *Enterobacteriaceae* phages in 20 MDD patients with high LPS levels. Long‐term preclinical studies (6 months in mice) show no adverse effects (e.g., phage‐induced inflammation), but risks include phage resistance (pathogens developing CRISPR‐based defenses) and unintended lysis of beneficial bacteria [57].

### 2.6. AI‐Driven Personalized Intervention Strategies

Machine learning integrates metagenomics and clinical phenotypes to model gut microbiota–depression links. MDD patients show reduced ratios of neuroprotective butyrate‐producing bacteria to proinflammatory bacteria, a potential typing marker [58]. Deep learning links glutamate synthase (gltB/gltD) and melatonin metabolism genes to *F. prausnitzii* abundance, providing a basis for microbiome‐based diagnostics. Machine learning models integrating multi‐omics data (metagenomics + metabolomics + neuroimaging) outperform clinical markers alone. For example, a model using the *Coprococcus*/*Dialister* ratio predicted probiotic response with AUC = 0.82 [59]. Wearable biosensors and AI enable real‐time therapeutic adjustments: A proof‐of‐concept study used smart capsules to monitor intestinal SCFAs, with reinforcement learning adjusting probiotic doses [37]. If SCFA levels drop below 5 *μ*mol/L (a threshold linked to symptom relapse), the AI system recommends a 20% increase in probiotic dose. The AI‐optimized group showed 17% greater HAM‐D reduction and more stable flora diversity than fixed‐dose groups [37].

However, small sample sizes (*n* = 30) and lack of validation in diverse populations (e.g., elderly and comorbid patients) hinder widespread use [37]. Long‐term data on algorithm accuracy is missing. Future multiomics studies should verify microbial metabolic pathway activity and cerebrospinal fluid neurotransmitter dynamics to optimize subtype identification.

### 2.7. Synthetic Biology–Modified Strains

Synthetic biology transforms probiotics into “living pharmaceutical factories.” *E. coli* Nissle 1917 engineered with the tryptophan hydroxylase gene converts dietary tryptophan to 5‐HTP, increasing brain 5‐HT and reducing forced swimming immobility in CUMS mice [36]. Similarly, *L. lactis* engineered to secrete BDNF enhances synaptic plasticity via TrkB receptor activation—reducing immobility time by 40% in CUMS mice, vs. 25% for unmodified *L. lactis* [35].

Engineered strains use attenuated vectors (deletion of virulence genes) and inducible expression systems to limit off‐target effects. However, they carry risks of horizontal gene transfer (e.g., antibiotic resistance genes) in immunocompromised patients [36]. Regulatory approval as “live biotherapeutic products” (LBPs) requires long‐term safety data (e.g., 5‐year follow‐up for gene stability).

### 2.8. Comparative Prioritization of Microbiota‐Targeted Interventions

To guide clinical application and future research, we comparatively rank the above interventions based on their current evidence base and near‐term clinical potential (Table 2).

**Table 2:** Comparative prioritization of microbiota‐targeted interventions.

**Intervention**	**Current evidence**	**Clinical potential**	**Key rationale**
Probiotics (conventional/postbiotics)	Abundant RCTs (e.g., [30, 34]) showing efficacy in MDD, postpartum depression; consistent preclinical mechanistic support	High	Noninvasive, favorable safety profile, scalable; postbiotics address viability limitations of live probiotics
Precision diet (high‐fiber/Mediterranean/anti‐inflammatory)	Strong observational and trial data (e.g., PREDIDEP [40]) linking to reduced depression risk; mechanistic validation via SCFAs	High	Low cost, accessible, synergistic with other interventions; personalized plans address heterogeneity
FMT	Preliminary human data in TRD [47]; robust preclinical efficacy [46]	Moderate	Potential for treatment‐resistant subgroups; limitations include microbiota reversion and donor standardization
Targeted antibiotics (minocycline/rifaximin)	RCT support in inflamed MDD subgroups [51, 53]; meta‐analytic confirmation for minocycline [50]	Moderate	Rapid anti‐inflammatory effects; use restricted to specific subtypes to avoid dysbiosis
AI‐driven personalization	Proof‐of‐concept studies [37]; multiomics prediction models [59]	Moderate–low	Addresses interindividual variability; requires larger validation cohorts and accessible biosensors
Phage therapy	Preclinical mechanistic data [56, 57]; upcoming pilot trial (NCT05876321)	Low	Strain‐specific targeting reduces off‐target effects; lacks human depression data
Synthetic biology–modified strains	Promising preclinical results [35, 36]	Low	High therapeutic potential; safety concerns (gene transfer, bacteremia) and regulatory barriers delay translation

*Note:* Ranking considers three core criteria: (1) quantity/quality of human clinical data (RCTs > observational studies > preclinical only); (2) safety profile (noninvasive/low risk > invasive/high risk); and (3) feasibility of implementation (scalable/accessible > resource‐intensive).

## 3. Challenges and Future Directions

### 3.1. Key Challenges

#### 3.1.1. Preclinical‐to‐Clinical Translational Gaps

A major barrier to translating MGB axis research to depression treatment lies in the disconnect between preclinical and clinical evidence. Most causal mechanistic data are derived from animal models, while human studies exhibit substantial heterogeneity, with core animal–human discrepancies driving this gap. Conventional laboratory mice have a gut microbiota dominated by *Bacteroidetes* (60%–70% relative abundance), nearly triple the proportion observed in humans (20%–30%) [22], and they lack key human commensal species such as *Akkermansia muciniphila*—a bacterium critical for intestinal barrier integrity and SCFA metabolism in human depression [32]—further limiting the translational relevance of mouse‐derived findings. Additionally, the widely used CUMS mouse model relies on acute, controllable stressors (e.g., cyclic food deprivation, cage tilting) to induce depressive‐like behaviors, whereas human MDD typically involves chronic, heterogeneous stressors (e.g., prolonged social adversity, traumatic life events) that interact with individual psychosocial contexts, creating a mismatch in the biological and psychological drivers of depression across species. Compounding this, mice possess a proportionally smaller prefrontal cortex (PFC)—a brain region central to emotion regulation, decision‐making, and MGB axis signaling in humans [60]—and this structural difference alters how gut‐derived signals (e.g., SCFAs and cytokines) modulate neural activity, weakening the translatability of mouse‐based neural mechanism findings. Beyond the MGB axis, inter‐organ communication pathways like the lung–brain axis also contribute to neuroimmune regulation and mental health, as the lung, a key immune organ, can mediate systemic inflammatory responses that impact CNS function—highlighting the need to consider multiaxis crosstalk in preclinical models to improve translational relevance [61].

#### 3.1.2. Drivers of Human Study Heterogeneity

Heterogeneity in human clinical trials of microbiota‐targeted interventions stems from four interrelated factors that undermine the consistency of efficacy outcomes. Genetic variability plays a key role, as polymorphisms in stress‐ and metabolism‐related genes shape intervention responses; for example, carriers of the 5‐HTTLPR s‐allele (linked to reduced serotonin transporter expression) are more sensitive to high‐sugar diets [44], leading to poorer outcomes with dietary interventions that do not account for this genetic trait. Baseline microbiota differences also directly impact efficacy: patients with low baseline abundance of the butyrate‐producing genus *Faecalibacterium* show significantly greater symptom improvement with probiotics [39], whereas those with high Bacteroidetes abundance (a taxon linked to systemic inflammation) derive minimal benefit from the same probiotic regimens [25]. Comorbid medical conditions further complicate results, as MDD frequently co‐occurs with metabolic or gastrointestinal disorders that alter MGB axis function—for instance, MDD patients with Type 2 diabetes exhibit a 40% lower response rate to FMT [48], attributed to impaired SCFA metabolism (a key mediator of FMT′s antidepressant effects) in the context of insulin resistance. Finally, inconsistent intervention protocols amplify heterogeneity, including differences in probiotic dosing (ranging from 10^9^ to 10^11^ CFU/day [30]) and FMT administration routes (oral capsules vs. colonoscopy [47])—both of which influence microbiota engraftment and clinical outcomes. To mitigate this heterogeneity, two strategies are proposed: using a “microbiota–inflammation–metabolite” panel (e.g., combining SCFA levels, CRP concentrations, and *Faecalibacterium* abundance) to stratify patients by biological subtypes, ensuring interventions are targeted to those most likely to respond; and prioritizing long‐term interventions (≥ 12 months) over short‐term studies (4–8 weeks), as gut microbiota composition stabilizes over time, minimizing the impact of transient fluctuations in flora on efficacy outcomes.

#### 3.1.3. Limitations, Risks, and Regulatory Considerations

Microbiota‐targeted interventions for depression face practical, safety, and regulatory challenges that must be addressed for clinical adoption. In terms of efficacy limitations, FMT is hindered by “microbiota reversion,” where 60% of patients return to their baseline gut microbiota composition within 12 months of treatment [48], eroding long‐term antidepressant effects; probiotics also show variable efficacy, with postintervention *Lactobacillus* abundance varying by 10‐fold across patients [30], likely due to interindividual differences in gut ecology. Safety risks are equally notable: broad‐spectrum antibiotics (e.g., minocycline) reduce gut microbiota diversity by 30% [52] and increase the risk of antibiotic resistance, while engineered probiotics carry unique hazards, including potential bacteremia in immunocompromised patients and horizontal transfer of engineered genes (e.g., antibiotic resistance cassettes) to commensal bacteria [36]. Regulatory and ethical hurdles further complicate progress: FMT lacks standardized global guidelines for donor screening, with criteria for excluding donors with *Clostridioides difficile* colonization varying between the FDA (United States) and EMA (EU) [48], and engineered probiotics require approval as LBPs—a regulatory pathway that mandates long‐term safety data (e.g., 5‐year follow‐up to assess gene stability), which is currently lacking for depression‐focused strains.

### 3.2. Translational Roadmap and Future Priorities

#### 3.2.1. Translational Roadmap

Table 3 outlines a phased approach to bridge preclinical research and clinical practice, with a focus on validating safety, optimizing efficacy, and ensuring long‐term sustainability. The preclinical stage prioritizes mechanism validation, involving testing microbiota‐targeted interventions in humanized gnotobiotic mice (colonized with human gut microbiota) to reduce species‐specific bias [22]. Phase I shifts to safety assessment, with small‐scale trials (*n* = 20–30) of engineered probiotics to evaluate risks of bacteremia and unintended gene transfer. Phase II focuses on efficacy stratification, designing randomized controlled trials (RCTs) that use “microbiota typing” to stratify patients, ensuring interventions are tested in biologically relevant subgroups (e.g., low SCFA/high CRP patients). Finally, Phase III emphasizes long‐term sustainability, implementing RCTs with ≥ 24 months of follow‐up to assess the durability of effects for FMT and probiotics, with a focus on preventing microbiota reversion.

**Table 3:** A phased approach to bridge preclinical research and clinical practice.

**Stage**	**Focus**	**Actions**
Preclinical	Mechanism validation	Test interventions in humanized mice (human microbiota transplanted) [22]
Phase I	Safety	Small trials (*n* = 20–30) of engineered probiotics to assess bacteremia risk
Phase II	Efficacy stratification	RCTs with microbiota/metabolome stratification (e.g., high vs. low CRP)
Phase III	Long‐term sustainability	Follow − up ≥ 24 months to assess FMT/probiotic durability [48]

#### 3.2.2. Priority Research Directions

To address the challenges outlined above, three priority research directions are identified to advance the field. First, validating mechanisms in human ex vivo models is critical—using 3D coculture systems (e.g., intestinal organoids paired with brain microvascular endothelial cells) to replicate MGB axis signaling in a human‐relevant context will help confirm animal‐derived mechanisms (e.g., SCFA‐mediated BBB protection) and reduce reliance on species‐mismatched data [60]. Second, developing clinically actionable “microbiota typing” tools is essential, as creating rapid, cost‐effective tests (e.g., PCR‐based assays to detect butyrate‐producing bacteria like *Faecalibacterium*) will enable routine patient stratification, ensuring interventions are directed to responders, reducing trial heterogeneity, and improving clinical outcomes. Third, testing combination therapies for sustained efficacy is a key focus, evaluating regimens that combine primary interventions with maintenance strategies to prevent microbiota reversion—for example, a pilot study demonstrated that supplementing FMT with *Akkermansia muciniphil*a reduced microbiota reversion by 60% at 12 months, supporting the potential of combination approaches to enhance long‐term sustainability [60].

## 4. Conclusions

The MGB axis has emerged as a therapeutic goldmine for depression, offering a paradigm shift from symptom management to etiological intervention. By decoding the complex interplay of neural, immune, and metabolic pathways and leveraging innovations in synthetic biology and AI, we stand on the cusp of a new era in mental health—one where personalized microbiome therapy becomes as routine as pharmacotherapy.

Notably, the comparative prioritization of microbiota‐targeted interventions highlights that probiotics and precision dietary approaches currently lead in clinical translatability, backed by robust human evidence and favorable safety profiles. In contrast, phage therapy and synthetic biology‐modified strains hold great promise but require further preclinical and clinical validation to address safety and feasibility barriers. FMT and targeted antibiotics occupy a middle ground, with utility in specific subgroups (e.g., TRD and inflamed phenotypes) but needing refinement to overcome microbiota reversion and dysbiosis risks.

However, critical challenges remain: preclinical‐to‐clinical translatability gaps, human study heterogeneity, and safety/regulatory hurdles. Addressing these requires interdisciplinary collaboration—combining microbiology, neuroscience, and data science to develop stratified, sustainable interventions. The promise of gut‐driven mental wellness is undeniable, but realizing it will depend on rigorous, patient‐centric research.

## Ethics Statement

The authors have nothing to report.

## Consent

The authors have nothing to report.

## Disclosure

All authors have read and approved the submitted version.

## Conflicts of Interest

The authors declare no conflicts of interest.

## Author Contributions

L.W. and Y.T. conceived the study, designed the research, and wrote the manuscript. L.Z., S.Z., and R.L. collected available data, conducted methodology, and analyzed data interpretation.

## Funding

This study was supported by the Educational Revolution Research Project in University Undergraduate Education of Hunan Province, 202401000348, and the Natural Science Foundation of Hunan Province, 2025JJ50483.

## Data Availability

Data sharing is not applicable to this article as no datasets were generated or analysed during the current study.
